# Prostatic Hypertrophy

**Published:** 1906-06

**Authors:** 


### SELECTIONS.

### Prostatic Hypertrophy.

(*Saint Louis Courier of Medicine.*)

WITHOUT question, one of the most important, most painful, and, at times, one of the most distressing affections to which men in advanced middle life are subject, is prostatic hypertrophy. That the disease is of not infrequent occurrence is evidenced by the fact that of 360 men examined by Johnson, 79 per cent. were afflicted with the enlargement of the prostate. It must be admitted, however, that only about 18 per cent of the sufferers present distressing symptoms. During the past few years our knowledge concerning prostatism has been markedly advanced, and in this brief résumé the recent findings will be discussed.

### ETIOLOGY.

The true etiology of prostatic hypertrophy has been, and continues to be, a much discussed question. John Hunter advanced the theory that the lesion was of inflammatory origin. Virchow and others concurred in this belief. The various other theories presented are:

1. That the lesion is a senile fibrotic change shared by other organs of the body.

2. That it is produced by sexual excess.

3. That it is due to ungratified sexual desire.

4. That it is a change secondary to degeneration of the bladder, and is an attempt to counteract the same.

5. That it is due to the action of the testes (preverted testicular secretion).

6. That it is a change normal to advanced years.

7. That it is due to chronic inflammation practically producing a cicatricial contraction, which ends in constriction of the gland acini or gland ducts.

8. That it is a catarrhal process of septic origin, which gains access from the bladder and urethra.

9. That it is a new growth of adenomatous nature.

Bangs is a strong adherent of the theory that the overaction and unphysiological—especially the latter—exercise of the prostate precedes, finally excites, and then prolongs the inflammatory irritation which eventuates in the recognised tissue changes. Of 300 cases of enlarged prostate examined by him, 85 per cent of the patients were subjects of abnormal or unphysiological sexual indulgences which were excessive and continued for years. Pilcher feels confident that it is not necessarily the length of functional activity of the gland and age of the individual which causes prostatic hypertrophy, but that it is a glandular overgrowth influenced by the degenerative changes of old age in an actively functionating gland which produces the change. A previous gonorrheal infection, or any other inflammatory process, may influence the development of the disease. It is worthy of mention that Cumston found that 80 per cent. of patients suffering from gleet show prostatic involvment. It naturally follows that it would be interesting to know how many of these latter cases develop chronic prostatic hypertrophy.

Wallace very correctly maintains that many of the theories advanced do not merit consideration. He feels confident that the change is an adenomatous one, the exciting cause of which may be chronic inflammation. Thayer concurs in the opinion that the lesion is due to long continued congestion, kept up for years. This may be the result of masturbation, imperfect intercourse, habitual excitment of the organ without the normal relief of the functional hyperemia consequent on ejaculation, extention of chronic inflammation from the posterior urethra, and varicose conditions of the veins and impeded return. It is thus quite evident that investigators are not universal in their opinion as to the etiology. That prostatic hypertrophy is of chronic inflammatory origin must be admitted. Just what the exciting factor of the latter may be, is the crux of contention.

PATHOLOGY.

Squier looks upon prostatic hypertrophy as an interstitial change which has been insidious in its onset, first affecting the innervation of the gland and its enclosed urethra, giving rise through its close connection with the hypogastric plexus of the sympathetic, the lumbar plexus, and spinal nerves, to many reflex symptoms in the kidney and bladder, as well as those of a purely sexual character, and eventually, with the declining vigor of the patient's later years, becoming a mechanical obstruction to the outlet of an already overworked bladder. Stoker describes three conditions of senile enlargement of the prostate:

1. True hypertrophy of the gland without any interstitial growths.

2. The existence upon and within the substance of the gland of one or more encapsulated tumors, distinct from the substance of the gland proper, and myomatous or adenomatous in structure. Both forms of neoplasm or only one of them may be present.

3. A mixed condition of true hypertrophy and interstitial growths of the second class.

This latter condition is much the more common. The tumors may eventually reduce the prostate to a state of pressure atrophy, or cause it to become a mere capsule for adventitious growth. Stoker maintains that it is this latter condition which in all probability has given rise to so much dispute, and uncertainty as to the true pathology of enlarged prostate.

Alexander has asserted that the so-called third lobe of the prostate is always of the nature of an overgrowth from one or other lateral lobe; enlargement of the prostate particularly affects the lateral lobes. He has found that the layer of prostate behind the urethra does not enlarge; the middle enlargement may be of three varieties: first, muscular, those cases with a bar; second, glandular, and then encapsulated; third, hypertrophy of mucous glands and tissue.

According to Pilcher's findings there are three types of prostates causing urinary obstruction:

1. The large, soft type.
2. The hard, small, contracted type.
3. The mixed type.

He contends that the contracted form is not a secondary stage of the large, soft type of the hypertrophy, but is distinct from it. He states that in some of the atrophic cases the glandular elements are relatively diminished and the muscular element relatively increased. Hypertrophy results from glandular overgrowth, influenced by the degenerative changes of old age, and other agents which tend to produce formation of fibrious connective tissue in an actively functionating gland. In many instances there is present a true muscular hypertrophy.

It has been stated that the histological examination indicates that the point of origin of the inflammatory process is in the prostatic urethra, extending thence along the gland ducts from the urethra toward the periphery of the prostate, the round-celled infiltration being most marked in the vicinity of the verumontanum. Bangs contends that the verumontanum is usually enlarged and tender in patients who practise coitus reservatus or coitus interruptous, and in those who have committed sexual excesses. Wallace believes that the usual form of prostatic hypertrophy is an adenomatous one, and that the adenomatous tissue may surround the urethra or form masses in the lateral parts of the organ, behind the urethra, or in all three parts.

In a very recent, and I may add a very excellent, monograph, Wallace says that all observers admit:

1. The changes do not occur before middle life.
2. The appearance in the normal spongy tissue of the opaque areas composed for the most part of gland tissue.
3. The growth of such areas either singly or in groups, to form, in a considerable number of cases, encapsulated tumors.
4. The preponderance in the stroma of such tumor of fibrous over muscular tissue.
5. The round celled infiltration.
6. The appearance in alveoli of desquamated epithelium cells, polymorphonuclear leukocytes and amyloid bodies.

Chetwood calls attention to the interesting fact that prostatism may be due to a contracture of he neck of he bladder.

Keyes states that the most notable changes associated with hypertrophy of the prostate are:

1. Bulging of the posterior surface.
2. Elevation of the urethral orifice.
3. Production of a middle lobe.
4. Lengthening and distortion of the prostatic urethra.

A future article will consider the symptomatology and diagnosis.—E. A. BABLER.

